# Increased female resistance to mating promotes the effect of mechanical constraints on latency to pair

**DOI:** 10.1002/ece3.4373

**Published:** 2018-07-30

**Authors:** Chang S. Han, Piotr G. Jablonski

**Affiliations:** ^1^ Laboratory of Behavioral Ecology and Evolution School of Biological Sciences Seoul National University Seoul South Korea

**Keywords:** female resistance, sexual conflict, size‐assortative mating, water strider

## Abstract

Size‐assortative mating, defined as a positive linear association of body size between members of mating pairs, can arise from mechanical constraints on pairing efficiency, particularly when mating success is affected by males’ mate‐grasping force. In this context, female resistance is predicted to have an important role in changing the threshold force necessary for males to hold females, thereby contributing to the effect of mechanical constraints. Thus, increased female resistance is expected to increase the paring success of an optimally sized male relative to the female body size (sexual size ratio = male body size/female body size = 0.86), which leads to positive size‐assortative mating. However, very little is known about the extent to which female resistance affects mechanical constraints on mate grasping. Here, using the water strider *Gerris gracilicornis* (Hemiptera: Gerridae), we tested whether the level of female resistance affected the relationship between the sexual size ratio and latency to pair. We found that optimally sized males mated sooner than other males when females resisted a male's mating attempts. When females did not resist, an effect of sexual size ratio on latency to pair was not found. Our results thus imply that increased female resistance to male mating attempts may strengthen the pattern of size‐assortative mating. We provide clear empirical evidence that female resistance to mating influences the effect of mechanical constraints on size‐assortative mating under sexual conflict. This result further suggests that patterns of size‐assortative mating can be altered by a variety of ecological circumstances that change female resistance to mating in many other animal species under sexual conflict.

## 1 INTRODUCTION

Size‐assortative mating, defined as a positive linear association of body size between members of mating pairs (“true” form of size‐assortative mating, (Arnqvist, Rowe, Krupa, & Sih, 1996)), can contribute to premating isolation between populations (Bolnick & Kirkpatrick, 2012; Coyne & Orr, 2004; Johannesson, Rolan‐Alvarez, & Ekendahl, 1995; Kondrashov & Shpak, 1998) and the evolution of sexual size dimorphism by favoring certain sexual size ratios (male/female body size ratio) (Han, Jablonski, Kim, & Park, 2010). Given the importance of the evolutionary consequences of size‐assortative mating, it is important to understand the causes that explain its occurrence. Although previous studies of size‐assortative mating have focused on adaptive causes such as a preference for a large mate (Arnqvist, 2011; Arnqvist et al., 1996), size‐assortative mating can occur in the absence of a mate choice mechanism (Arnqvist et al., 1996; Bollache & Cézilly, 2004; Crespi, 1989; Galipaud, Bollache, & Dechaume‐Moncharmont, 2013; Han et al., 2010; Jiang, Bolnick, & Kirkpatrick, 2013).

**Figure 1:**
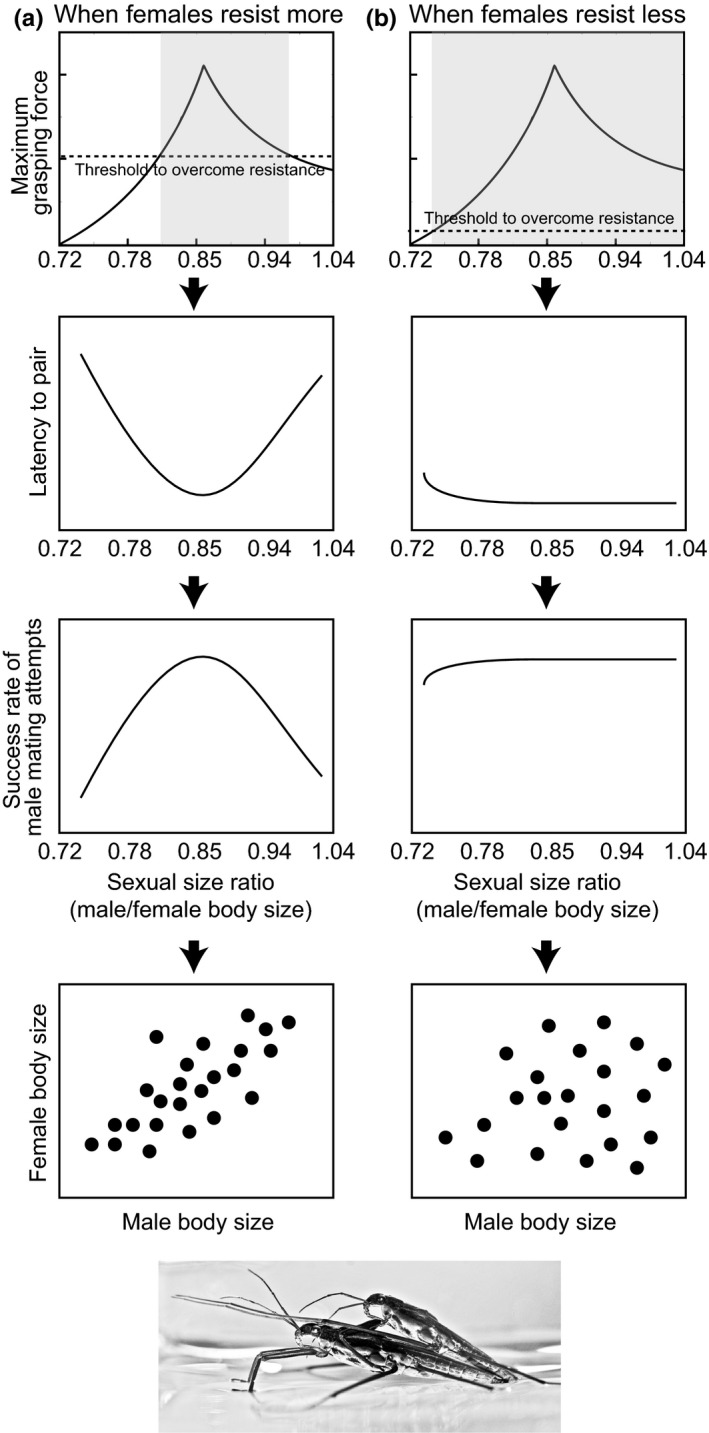
Relationship between a range of male body size relative to female body size (sexual size ratio) and threshold force (dotted lines) necessary for males to successfully grasp females at the initiation of mating. Shaded areas indicate a range of body sizes of males relative to females where the maximum grasping force is greater than the threshold and where the initiation of mating attempts is successful. (a) The threshold force is high when females resist more, and there is a narrowing of the optimal male size range (relative to a certain female size) that leads to a successful initiation of mating. This is expected to result in a strong size‐assortative mating pattern. (b) The threshold force is low when females resist less, widening the range of optimal relative male size for the successful initiation of mate grasping. This is expected to result in a weak size‐assortative mating pattern

Mechanical constraints on paring can result in size‐assortative mating in species in which a male grasps a female and overcomes the female's resistance to mating in order to attempt to mate successfully (Crespi, 1989; Han et al., 2010) (Figure 1). In these species, a male is unable to pair with a female that is too large or too small relative to his size. Mismatched males are not able to produce grasping forces that are large enough to overcome the female's resistance to mating and do not achieve successful mating. Thus, size‐assortative mating can arise from mechanical constraints on pairing, particularly when males must produce strong grasping forces to successfully pair with females (Crespi, 1989; Han et al., 2010) (Figure 1).

A recent mechanical model suggested that physical constraints on mate grasping account for the size‐assortative mating pattern in water striders (Heteroptera: Gerridae) (Han et al., 2010). Mating by water striders is initiated when the male forcefully mounts a female. At the initiation of the mating attempt, the mounting male tries to tightly grasp the female's thorax with his forelegs, overcome female resistance, and remain on top of the female (Figure 1). Males can have successful mating attempts only when they are able to produce grasping forces strong enough to hold females during the struggling that occurs at the initiation of mating. Our previous theoretical model showed that the optimal size of a male for producing the strongest grasping force is 0.86 relative to the female body size (Han et al., 2010). When relative male body size is optimal for mate grasping, the male would have a shorter latency to pair than that for other males who are not with the optimal sexual size ratio range for mate grasping (Figure 1, Han et al., 2010).

In the mate‐grasping mechanical model (Han et al., 2010), female resistance is predicted to be an important factor that determines the effect of mechanical constraints on size‐assortative mating. For females that resist less than others, the threshold force necessary for males to hold onto them decreases (Figure 1b). Thus, the optimal male size range (relative to a certain female size) increases, indicating that the effect of grasping mechanics on male mating success and size‐assortative mating decreases. In contrast, increased levels of female resistance cause an increase in threshold force needed to overcome female resistance (Figure 1a). In this case, there is a narrower range of optimal male size for mate grasping, which increases the effect of mate‐grasping mechanics on male mating success. As a result, the strength of size‐assortative mating is predicted to increase. Despite its theoretical possibility, no studies have empirically examined whether female resistance to mating attempts changes the effect of mechanical constraints in mate grasping on size‐assortative mating.

The Korean water strider *Gerris gracilicornis* (Heteroptera: Gerridae) offers an ideal system in which to understand the relationship between female resistance to mating and size‐assortative mating. Mate‐grasping mechanics leading to size‐assortative mating of *G. gracilicornis* has been supported by a theoretical model and empirical evidence (Han et al., 2010). Once a male *G. gracilicornis* mounts a female and initiates copulation, the female becomes less resistant and the male can remain mounted for up to 2 days (CS Han, personal observation). However, when the male fails to grasp the female firmly and copulation is delayed, other single males nearby may interfere with the first male's attempts to grasp the female, and an interfering male can sometimes take over the female (e.g., Han & Brooks, 2013b). Thus, rapid and successful mate grasping is important for the reproductive success of male *G. gracilicornis*. In this study, we measured the initial phase of pair formation (latency to pair) for different sexual size ratios (male to female body size ratio) in mating pairs when males attempted to copulate with females. Then, we assessed whether female resistance altered the relationship between sexual size ratio and a male's latency to pair. We predicted that the degree of deviation from the optimal sexual size ratio of a pair (M/F = 0.86) determined a male's latency to pair more strongly when females resisted male mating attempts.

## 2 MATERIALS AND METHODS

### 2.1 Mate grasping of male G. gracilicornis

The precopulatory phase for male *G. gracilicornis* is divided into several steps (Han & Jablonski, 2009). First, a male grasps the female and tries to align his body parallel to her body. The male then attaches his genitalia to the surface of a female gonocoxae (genitalia). At last, the mounting male produces courtship signals until the female protrudes her ovipositor and the male's genitalia grab hold of it (successful intromission). Of these steps, the period from the first grasping to aligning his body parallel to her body (latency to pair) is predicted to be the most dependent on the mechanical constraints on mate grasping (Han et al., 2010). During this period, because of the instability of the forelegs’ grip, a male is vulnerable to a female's attempts to dislodge him. If the male releases his forelegs from the female's body when the female tries to push him away, he can be easily thrown off. Thus, males outside the optimal sexual size ratio (male size relative to female size) range are predicted to be unable to produce grasping forces strong enough to counter female resistance to mating. In addition, when the male fails to grasp the female firmly, he is unable to attach his genitalia to her genitalia while holding the female. When copulation is delayed, other single males nearby may interfere with the first male's attempts to grasp the female, and another male can take over the female (CS Han, personal observation). Because males that successfully mount a female can stay mounted for up to 2 days (CS Han, personal observation), successful and rapid mate grasping guarantees the long‐term reproductive success of male *G. gracilicornis*. Thus, mechanistic constraints determine the success of mate grasping and thereby contribute to the pattern of size‐assortative mating (Han et al., 2010). In this study, we measured the time males spent grasping and aligning their body (latency to pair) to test the role of mate‐grasping mechanics in size‐assortative mating.

### 2.2 Rearing conditions and experiment

Individual *Gerris gracilicornis* used in this experiment were collected at Gwanak Mountain near Seoul National University, Seoul, South Korea. In the laboratory, water striders were fed ad libitum with a surplus of frozen crickets (*Gryllus bimaculatus*) every 2 days. Pieces of floating Styrofoam were used as rest sites for the water striders.

Under laboratory conditions, we closely observed 110 pairs to measure the effect of a pair's sexual size ratio on males’ latency to pair. For the tests, we put a male and a female, which were randomly selected and had been isolated from the opposite sex for 3 days, in a transparent experimental basin (15 × 30 cm, water depth 5 cm) and observed their behavior. We measured (a) the latency to pair (duration (in seconds) from the first grasp to the male aligning his body parallel to the female's body), (b) whether the female resisted during the period, and (c) the sexual size ratio (male body size/female body size) of the pair. The body size of water striders was measured with the ImageJ program (National Institutes of Health, USA) after taking digital photographs of the ventral surface of each individual.

The latency to pair is highly dependent on the mechanical constraints related to mate grasping (Han et al., 2010) and the most important factor determining the premating success of males (Supporting Information Appendix S1). During this period, because of the instability of the forelegs’ grip, a male becomes vulnerable to a female's attempt to dislodge him. If the male releases his forelegs from the female's body when the female tries to push him away, he can be easily thrown off. Thus, males outside the range of the optimal sexual size ratio (male size relative to female size) are unlikely to be able to produce grasping forces strong enough to counter the female's resistance and are likely to fail in their attempt to mate (Supporting Information Appendix S1).

### 2.3 Statistical procedures

We used a general linear model, where the square‐root‐transformed and (mean and variance) standardized latency to pair were fitted as the response variable and where the z‐transformed sexual size ratio (M/F) of a each pair (covariate), its quadratic term, female resistance to mating (two‐level factor: absence versus presence), and their interactions were fitted as fixed factors. In addition, to measure the effect of the sexual size ratio on the level of female resistance, we used a generalized linear model, where the female resistance was fitted as the response variable and where the z‐transformed sexual size ratio was fitted as a covariate. All analyses were performed using the Statistica 10 (StatSoft Inc.).

## 3 RESULTS

**Figure 2:**
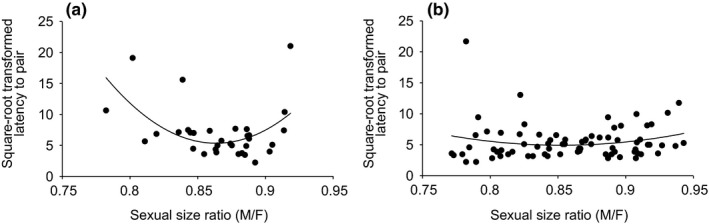
Effect of female resistance on the relationship between sexual size ratio (M/F) and latency to pair. (a) The presence (n = 39) or (b) absence (n = 71) of female resistance to mating

The effect of sexual size ratio on the latency to pair strongly depended on the level of female resistance to mating (effect of sexual size ratio^2^ × resistance, Table 1, Figure 2). When females showed resistance to mating, males smaller or larger than the optimal sexual size ratio (M/F = 0.86) had a much longer latency to pair (Figure 2a). However, latency to pair was not affected by the sexual size ratio when females did not resist (Figure 2b). In addition, when we tested whether females preferred males that were relatively smaller or larger, we found that female resistance did not differ across different sexual size ratios (Wald stat = 0.56, *df* = 1, *p* = 0.45).

**Table 1:** General linear model of the latency to pair as a function of female resistance and sexual size ratio

Variables	Coefficient (*SE*)	*t*	*p*
Intercept	−0.23 (0.13)	−1.75	0.08
Resistance	−0.05 (0.12)	−0.46	0.64
Sexual size ratio	−0.004 (0.12)	−0.04	0.97
Sexual size ratio^2^	0.55 (0.13)	4.13	<0.001
Sexual size ratio × Resistance	0.06 (0.12)	0.50	0.62
Sexual size ratio^2^ × Resistance	−0.50 (0.16)	−3.08	0.003

## 4 DISCUSSION

We found that female resistance to mating increased the mechanical constraints on mate grasping, which possibly resulted in stronger size‐assortative mating patterns (Table 1, Figure 2). Thus, our results provide support for the mechanical constraint model (Han et al., 2010). We predicted that female resistance affected how a mounting male efficiently grasps a female until copulation. Our results then showed that female resistance significantly increased the latency to pair for males outside of the optimal sexual size ratio range, which possibly increased the likelihood of failure in their mating attempts and consequently shaped a strong size‐assortative mating pattern (Figures 1, 2). In addition, given the similar levels of female resistance across sexual size ratios, female preference did not contribute to the size‐assortative mating pattern of *G. gracilicornis*. Therefore, we conclude that the size‐assortative mating pattern of *G. gracilicornis* was not caused by female preference but instead by the mechanical constraints on mate‐grasping efficiency.

In this study, we measured a males’ latency to pair and used these data to represent male reproductive success. However, because water strider males copulate with multiple females (Andersen, 1994; Arnqvist, 1997; Spence & Andersen, 1994), male reproductive success also depends on postcopulation sperm competition and on copulation with other females (Arnqvist, 1988, 1989; Arnqvist & Danielsson, 1999; Danielsson, 2001; Rubenstein, 1989), which may decrease the importance of mate grasping in male reproductive success. However, in *G. gracilicornis*, when a mounting male fails to grasp a female firmly, he cannot align his body and rapidly initiate copulation. As the latency to pair is increased, other single males nearby can approach the pair and interfere with the first male's mating attempt (CS Han, personal observation). This inference by other single males sometimes leads to a takeover and the female mates with interfering male. Courtship interference by other males is particularly common in water strider species that produce courtship ripple signals (Han & Brooks, 2013b). In addition, once a male *G. gracilicornis* mounts and copulates with a female, he can stay on her for up to 2 days, resulting in prolonged postcopulation mate guarding (Han et al., 2010), and during this period, he repeatedly copulates with her (CS Han, personal observation). In water strider species characterized by prolonged postcopulation mate guarding, a male's mate‐grasping success at the initiation of mating is strongly related to the short‐term (1 week) and long‐term (4 weeks) reproductive success of males (Han & Brooks, 2013a, 2014), which indicates that a shorter latency to pair for *G. gracilicornis* males is likely to reflect their lifetime reproductive success. In addition, because water striders characterized by prolonged postcopulation mate guarding also have a male‐biased sex ratio (Han & Brooks, 2013b), most females are occupied by males during the reproductive period, and therefore, the failure of a male in a mating attempt indicates that the male may not have a chance to mate for many hours or days. Although our results did not include events during courtship or postcopulation sperm competition, the latency to pair measured in our study is likely to be an important determinant of male reproductive success in *G. gracilicornis*. Female resistance increased the latency to pair and thereby increased the likelihood of mating failure for males outside of the optimal sexual size ratio range, which results in a strongly positive size‐assortative mating pattern.

A relationship between female resistance and patterns of size‐assortative mating is also predicted to be present in other animal species that utilize coercive mate‐grasping mechanisms in the precopulation period. For example, in crustacean *Gammarus* species (Amphipoda), males hold and guard females during the precopulatory period (Borowsky, 1984). One of the suggested reasons that shape their size‐assortative mating patterns is the loading constraint imposed when a male is carrying his mate (Adams & Greenwood, 1983; Williams, 2007). Furthermore, given the existence of female resistance during mate pairing in this species (Jormalainen, Tuomi, & Yamamura, 1994), we predict that patterns of size‐assortative mating for *Gammarus* males may depend on the level of female resistance to mating. Therefore, given that mate grasping with forelegs, midlegs, or even antennae is widespread in animals (reviewed in Eberhard, 1985), the relationship among female resistance, mate‐grasping constraints, and size‐assortative mating can be tested by studying other animal species.

Our results imply that ecological factors can affect patterns of size‐assortative mating. For example, in the case of water striders, females are generally more reluctant to mate in an environment with a female‐biased sex ratio to avoid superfluous mating (Rowe, 1992; Thornhill & Alcock, 1983; Wilcox, 1984). In addition, because resistance to mating is energetically costly to females, satiated females are able to engage in longer and more vigorous premating struggles with males than hungry females (Rowe, Arnqvist, Sih, & Krupa, 1994). Predation risk is also known to decrease (Han & Jablonski, 2010) or increase female resistance to mating (Sih & Krupa, 1992). Thus, when ecological factors such as sex ratio, availability of potential prey or predation risks affect the level of female resistance to mating, they could also affect the strength of size‐assortative mating.

Furthermore, ecological conditions that change the level of female resistance and the strength of size‐assortative mating may also explain variations in sexual size dimorphism across populations in water striders. Strongly positive size‐assortative mating can exert stabilizing selection on male and female size distributions and can contribute to the evolution and maintenance of sexual dimorphism (Han et al., 2010). In an ecological condition where female resistance increases and the role of mate grasping in pairing success is important, strong size‐assortative mating shifts the level of sexual size dimorphism of water striders to a certain optimal sexual size ratio for males to be effective in mate grasping (Figure 6 in Han et al., 2010). In contrast, in an ecological condition where female resistance decreases and mate grasping is not important in male mating success, the level of sexual size dimorphism in water striders would be determined by processes other than optimal mate grasping (Figure 6 in Han et al., 2010). Therefore, an interesting avenue for future research would be to address the relationship among ecological conditions, female resistance, size‐assortative mating, and sexual size dimorphism.

Our study suggests that a change in female resistance to mating affects the degree of size‐assortative mating in a species where male premating success depends on mechanical constraints on the efficiency of mate grasping. We further suggest that it would be fruitful to test whether the strength of size‐assortative mating is influenced by a variety of ecological circumstances in other animal species that experience strong sexual conflict during the precopulatory period.

## CONFLICT OF INTEREST

The authors declare that they have no competing interests.

## AUTHOR CONTRIBUTION

CSH and PGJ conceived the study. CSH collected the data and carried out statistical analyses. All authors substantially contributed to drafting the manuscript and gave final approval for the publication.
