# Supplementary material for: Increased female resistance to mating promotes the effect of mechanical constraints on latency to pair
Source: Ecol Evol. 2018 Jul 30;8(18):9152–7. doi: 10.1002/ece3.4373 (PMC6194263; doi:10.1002/ece3.4373)
Supplement: Supplementary file 1 [file ECE3-8-9152-s001.docx]

**Appendix S1. Relationship between the latency to pair and initial mating success**

We observed pairs of water striders to assess if longer latency to pair led to failures of male mating attempts. We scored a male mate-grasping attempt as ‘successful’ when the mounting male abdomen tip was firmly pressed (attached) against the female abdomen’s tip (see Figure 1). We also scored an ‘unsuccessful’ attempt when the mounting male was dislodged by female resistance before the moment of successful genitalia attachment. In trials, no males dismounted voluntarily when females did not resist. Thus all the unsuccessful attempts were due to female resistance. To test the relationship between the latency to pair and initial mating success, we used a generalized linear model with binomial error and logit link function, where mating attempt success (successful/unsuccessful attempt, binary variables) was fitted as the response variable, and where latency to pair was fitted as a covariate. From the experiment, we found that increased latency to pair decreased success in males’ first mating attempt (N=110, estimate(±s.e.)=0.005(±0.002), P=0.02).
